# Plasma-Activated Water Produced by a Moderately High Energy-Efficient 1-Liter Reactor: Effects on Germination and Growth of Tomato and Bell Pepper Plants

**DOI:** 10.3390/plants14050722

**Published:** 2025-02-27

**Authors:** Matías G. Ferreyra, María M. Caffaro, Brenda Santamaría, Carla Zilli, Alejandra Hernández, Brenda L. Fina, Ada S. Vélez, Karina B. Balestrasse, Leandro Prevosto

**Affiliations:** 1 Grupo de Descargas Eléctricas, Departamento Ing. Electromecánica, Facultad Regional Venado Tuerto, Universidad Tecnológica Nacional, CONICET, Laprida 651, Venado Tuerto S2600, Argentina; brendasantamaria1989@gmail.com (B.S.); bfina@frvt.utn.edu.ar (B.L.F.); leandroprevosto@gmail.com (L.P.); 2 Instituto de Investigaciones en Biociencias Agrícolas y Ambientales, Facultad de Agronomía, Universidad de Buenos Aires, CONICET, Av. San Martín 4453, Ciudad Autónoma de Buenos Aires C1417DSE, Argentina; caffaro@agro.uba.ar (M.M.C.); czilli@agro.uba.ar (C.Z.); hernande@agro.uba.ar (A.H.); kbale@agro.uba.ar (K.B.B.); 3 Cátedra de Fertilidad y Fertilizantes, Facultad de Agronomía, Universidad de Buenos Aires, Av. San Martín 4453, Ciudad Autónoma de Buenos Aires C1417DSE, Argentina; 4 Cátedra de Bioquímica, Facultad de Agronomía, Universidad de Buenos Aires, Av. San Martín 4453, Ciudad Autónoma de Buenos Aires C1417DSE, Argentina; 5 Escuela para Graduados, Facultad de Ciencias Agropecuarias, Universidad Nacional de Córdoba, Ing. Agr. Felix Aldo Marrone N° 746, Córdoba X5000, Argentina; silvanavelez748@gmail.com

**Keywords:** plasma-activated water, non-thermal plasma, glow-type discharge, energy efficiency nitrogen synthesis, seed germination, plant growth, tomato, bell pepper

## Abstract

Plasma-activated water (PAW) is a sustainable and innovative alternative for agriculture, especially in controlled environments like greenhouses. Tomato and pepper are key horticultural crops worldwide, with a considerable part of their production in greenhouses. This study examined the effects of PAW irrigation on seed germination, plant growth, and oxidative stress in tomato and bell pepper plants. PAW was activated for up to 15 min using a 1 L capacity plasma reactor based on a glow-type discharge in air with water-cathode. The concentration of nitrogen compounds and the energy efficiency of synthesis obtained with the reactor were moderately high (5.4 mM and 60 nmol/J, respectively). The most notable effects of PAW were observed in bell pepper. The germination percentage in bell pepper increased by up to 26%, while no significant effects were found in tomato seeds. PAW irrigation significantly promoted plant growth, with dry weight increasing by up to 61% in bell pepper and 42% in tomato. Lipid peroxidation results showed no oxidative damage in either crop. The biochemical analysis of antioxidant enzymes (catalase, superoxide dismutase, and guaiacol peroxidase) confirmed that plant defense systems responded adequately to PAW irrigation. These results highlight PAW’s potential as an innovative and eco-friendly alternative in agriculture.

## 1. Introduction

In recent decades, a great deal of research on non-thermal electrical discharges has been dedicated to the study of their technological applications, such as materials, environmental care, materials, among others. One of the most explored areas is that known as ‘Plasmas in Agriculture’, on which an estimated 2000 scientific articles have been published annually in recent years [1]. Plasma applications in agriculture range from pre-harvest to post-harvest, and include everything from seed and food disinfection/decontamination to crop fertilization and plant growth [2].

Non-thermal plasma treatments in agriculture are divided into two categories according to their mode of application: “direct plasma treatment” or “indirect plasma treatment”. In direct treatment, substrates, such as seeds, are directly exposed to plasma. As a result, the substrates are exposed to intense electric fields, UV radiation, and reactive plasma particles, such as electrons, ions, or excited neutral particles. In indirect treatment, the substrates are soaked with liquids that have previously been exposed to plasma. This is one of the most recent techniques, and the activated liquids serve, for example, as a decontamination medium, or as a source of nutrients to stimulate seed germination and plant growth [2,3,4].

Within indirect treatment, one of the most used liquids is water. Water exposed to plasma is known as plasma ‘activated’ water (PAW) [5,6]. When water is exposed to a discharge in air or similar mixtures, reactive oxygen and nitrogen species (RONS) generated in the gas enter the water by diffusion based on their ability to solubilize in the liquid. The long-half-life species that can survive in liquid are nitrate (NO_3_^−^), nitrite (NO_2_^−^), and hydrogen peroxide (H_2_O_2_). These species are of great importance in agriculture. Plasmas in the air are presented as an alternative method of RONS synthesis in water with low energy cost and low complexity for technological implementation compared to traditional processes, such as the Haber–Bosch process [4,7].

RONS play an important role in the germination process [4,6,8]. Reactive oxygen species (ROS) act as signaling molecules that trigger events related to seed germination. H_2_O_2_ accumulation can influence hormone balance by increasing gibberellin (GA) and decreasing abscisic acid (ABA) and ethylene. This reorganization of hormone signaling can lead to the restarting of metabolic activity, which is essential for seed germination [6,9]. The NO radical is also a key signaling molecule involved in the release of seed dormancy. These radicals, although not found in PAW owing to their low solubility in the gas phase during the activation process, can be biologically generated in PAW-embedded seeds through nitrate and nitrite reductases [6,10]. Plant growth is directly related to the availability of macronutrients and micronutrients. Nitrogen (N) is the main macronutrient for plants, which explains why N deficiency is a growth-limiting factor. Reactive nitrogen species (RNS) are essential for plant nutrition by regulating the synthesis of proteins, amino acids, and chlorophyll. NO_3_^−^ is the main source of N for most plants, although NO_2_^−^ is also a source of N, especially in situations with low NO_3_^−^ levels. NO_2_^−^ can be toxic to plants, although some are tolerant and can metabolize this species, as is the case with tomatoes. For utilization by the plant, both the assimilated NO_3_^−^ and NO_2_^−^ begin their reduction process until they are converted into ammonium (NH_4_^+^), a compound used for the synthesis of amino acids. Both NO_3_^−^ and NO_2_^−^ species have been reported to act as signaling molecules, for regulating either plant growth and development or N homeostasis [4,11]. Likewise, studies also show that ROS, including H_2_O_2_, act as signaling molecules in plants and control growth by regulating stress responses [4]. For these reasons, PAW, as a solution with an appreciable amount of RONS, could be an alternative technique with great potential in agriculture, being also eco-friendly since it does not require the addition of chemical compounds.

Tomato and pepper belong to the Solanaceae family. Both are among the most important horticultural crops in the world. In Argentina, a significant percentage of tomato and pepper is produced in greenhouses. Tomato is the second most important horticultural crop in terms of production (one million tons per year) after potatoes. The cultivated area of tomatoes is about 10 thousand hectares, among which approximately 25% are within greenhouses [12]. Pepper cultivation in Argentina occupies approximately 6500 hectares, among which 1700 correspond to greenhouses, representing 30% of the horticultural area grown in greenhouses, only below that of tomatoes [13]. Under controlled crop growth conditions, as occurs with tomato and pepper in greenhouses, irrigation with PAW is an attractive option to stimulate plant development.

In recent years, some researchers have evaluated the effects of PAW on the germination and development of tomato and pepper plants [14,15,16,17,18,19,20]. Most of these studies have focused on evaluating the effects of PAW on plant development. The influence of PAW irrigation on tomato plant development was investigated, and increased plant length and weight [18,19]. The growth of pepper plants during the reproductive stage and the quality of their fruits have been evaluated [17]. Plants were grown from seeds soaked for 24 h in PAW, both in the field and greenhouse. The best results for both growth and fruit quality were obtained for plants grown in a greenhouse using PAW-treated seeds. The combined effect of the direct plasma treatment of tomato and pepper seeds followed by irrigation with PAW has also been investigated [20]. The results showed that tomato plants grown from non-plasma-treated seeds and irrigated with PAW or non-activated water grew similarly, whereas plasma-treated seeds irrigated with PAW showed an increase in plant development. The results in pepper, however, showed that, in plants grown from non-treated seeds, higher growth was observed for plants irrigated with PAW than for those irrigated with non-activated water, and even higher growth was observed for seeds treated with direct plasma and irrigated with PAW.

PAW can potentially increase oxidative stress due to its appreciable content of RONS, which may cause plant damage. Despite this, only a few studies have investigated the effects of PAW irrigation on plant growth and defense responses in tomatoes [14,15] and peppers [16]. These studies employed various discharge types, such as DBD, jets, and glow-type discharges, with high-frequency power supplies (kHz to MHz) [14,15,16,17,18,19,20]. Although all are classified as non-thermal plasmas, significant differences exist in their gas temperatures and chemical compositions, which in turn lead to significant variations in the composition of the resulting PAW.

One critical parameter in PAW production is the energy efficiency of RONS synthesis, as non-thermal plasmas in contact with liquids are considered a promising method of RONS synthesis [7]. However, this parameter is often overlooked, despite being easily derivable from plasma parameters and RONS concentrations. Given the scarcity of studies examining PAW effects on tomato and pepper plants—only seven studies published so far [14,15,16,17,18,19,20] among the 2000 annual publications in the “Plasmas in Agriculture” field [1]—the present work is an effort to provide further insight into these issues. Specifically, it evaluates PAW’s impact on plant defense mechanisms and the energy efficiency of the RONS synthesis process. Notably, this study achieved some of the highest RONS concentrations reported to date, with RNS (NO_X_ = NO_2_^−^ + NO_3_^−^) concentrations being 3–22 times higher than those reported in previous studies [14,15,16,17,18,19,20].

In this work, the effects of PAW on the germination and growth of tomato and bell pepper plants were determined. PAW was activated using a 1 L capacity plasma reactor based on a glow-type discharge in atmospheric pressure air with a water-cathode operated at quasi-stationary frequency (50 Hz). The maximum activation time of the PAW was only 15 min. The energy efficiency of the RONS synthesis process and the mass of RNS produced in water were estimated. The germination percentage and water absorption of the seeds were analyzed. In plants, the biometric growth parameters and chlorophyll content in the vegetative phase were determined. In addition, the oxidative damage in plants was determined through lipid peroxidation and antioxidant enzyme activity.

## 2. Materials and Methods

### 2.1. Plasma-Activated Water Reactor

**Figure 1:**
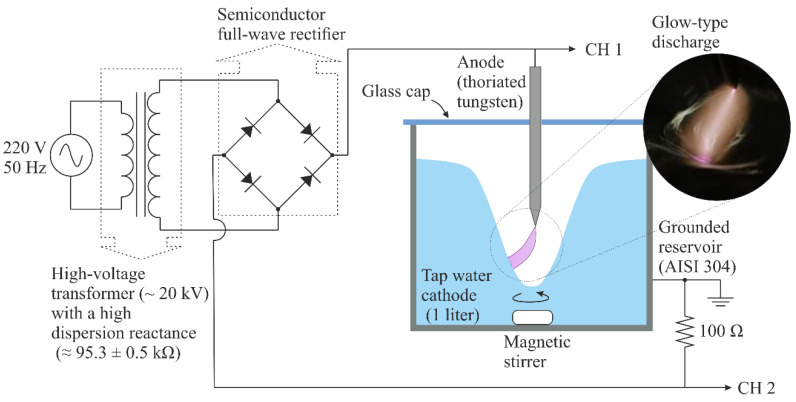
Schematic of the experimental setup to generate plasma-activated water with a non-thermal glow-type discharge in the atmospheric pressure air with a water-cathode.

A glow-type discharge in atmospheric pressure air with a water-cathode was used to generate PAW. A schematic diagram of the reactor is shown in Figure 1. The chosen electrode configuration was of the tip-to-water type, with the anode being a needle-shaped electrode (radius ~200 μm), constructed from a thoriated tungsten rod, and the cathode being the tap water to be activated (1 L). Water was contained in a grounded reservoir constructed in stainless steel (AISI 304) with a total volume of ≈1330 cm^3^. The separation between the electrodes (gap) was ~10 mm. A glass cap was placed on top of the reservoir to create a confined gas chamber. The purpose of having a confined gas chamber was to avoid the RONS generated by the discharge diffusing into the environment, thus improving RONS diffusion into the liquid. The volume of the gas confined in the chamber was about 330 cm^3^.

During the water activation process, a vortex was generated with a magnetic stirrer to increase the gas–liquid exchange surface and improve the mixing of the RONS entering the liquid from the gas phase. Another purpose of the vortex was to avoid excessive heating and the subsequent evaporation of water at the cathodic root, which is the point of contact between the water and plasma. In the same sense, and given the thermal fragility of H_2_O_2_, the activation reservoir was contained in a refrigerated water bath system during the activation process so that the temperature of the bulk of the activated water did not exceed 20 °C [21]. There were no significant variations in the water volume (<5 mL) after activation.

**Figure 2:**
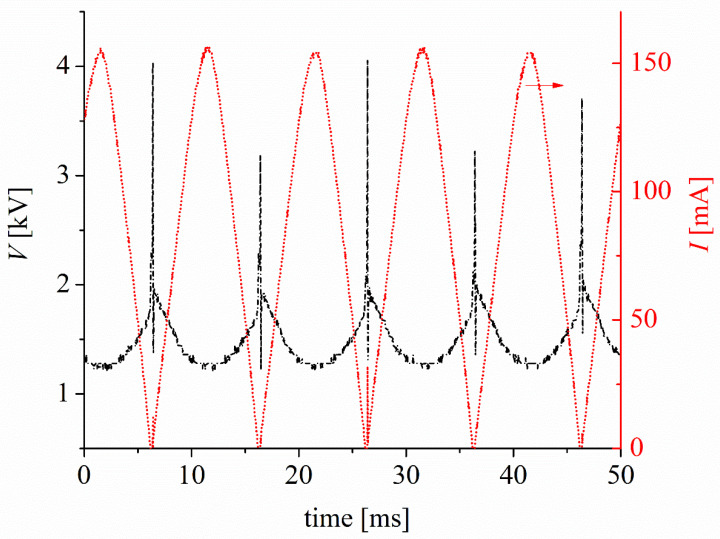
Voltage (*V*) and current discharge (*I*) waveforms of the glow-type discharge in atmospheric pressure air with the water-cathode. The discharge voltage waveform is black and the discharge current waveform is red. The arrow (above the discharge current waveform, in red) indicates that the corresponding axis for reading the discharge current value is the right axis. Therefore, the left axis corresponds to the discharge voltage waveform.

The discharge was ignited using a variable autotransformer to adjust the discharge current. This autotransformer was connected to the primary circuit of a high-voltage AC power transformer (~20 kV, 50 Hz) with a high dispersion reactance (95.3 ± 0.5 kΩ). This high impedance creates negative feedback between the discharge current and voltage without the need for an external ballast, eliminating the possibility of discharge transitioning to a high-current thermal discharge. A full-wave semiconductor bridge rectifier is used in the secondary circuit of the high-voltage transformer to fix the polarity of the electrodes. The discharge voltage was measured using a high-voltage probe (Tektronix P6015A, 1000X, 3 pF, 100 MΩ) connected to Channel 1 (CH1) of an oscilloscope (Tektronix TDS 2004C) with a sampling rate of 1 GS/s and an analog bandwidth of 70 MHz. Simultaneously, the discharge current was inferred by employing a shunt resistor (low inductance) of 100 Ω connected to Channel 2 (CH2) of the oscilloscope. Figure 2 shows the voltage (*V*) and current (*I*) waveforms of the discharge. The current signal exhibits an almost sinusoidal shape with a peak value of approximately 155 mA, which is controlled by the high dispersion reactance of the high-voltage transformer. The RMS value of the current pulse is 100 mA. At the beginning of each pulse of the voltage signal, voltage spikes of approximately 4 kV are observed. These spikes correspond to discharge ignition via a streamer-to-spark high-voltage transition [22]. After the breakdown, the voltage dropped to approximately 1 kV, owing to the high impedance of the transformer, and the discharge stabilized to a glow-type discharge. The measured voltage includes, in addition to the voltage drop in the gas gap, the voltage drops in the electrode non-neutral sheaths (mainly in the cathode sheath), as well as in the resistive cathode, that is, tap water. However, because the electrical conductivity of tap water is high (≈1600 µS/cm), the voltage drop in the liquid cathode is low compared to the voltage drop in the gas gap; thus, the measured voltage is approximately equal to the voltage drop of the discharge. The mean power *P* of the discharge is calculated as follows:

(1) $$P=\frac{1}{\tau }\;\int_{0}^{\tau }I\left(t\right)V\left(t\right)dt$$

where *τ* denotes the signal period. Under the aforementioned experimental conditions, *P* ≈ 100 W.

### 2.2. Physicochemical Properties of Plasma-Activated Water

A pH meter (Ohaus SP2200F, range of 0–14 and resolution of 0.01) and conductivity meter (Oakton Cyberscan Cond 610, range of 0–500 mS/cm, and accuracy of 1%) were used to determine the pH and electrical conductivity, respectively. The instruments were calibrated before the measurements using standard solutions with pH 7 and pH 10 buffers for the pH meter and a 0.01 M KI solution (electrical conductivity = 1413 μS/cm at 25 °C) for the conductivity meter. The concentrations of RONS (NO_2_^−^, NO_3_^−^, and H_2_O_2_) in the PAW were determined using the colorimetric methods reported by [23] using a UV–VIS spectrophotometer (Spectrum SP-2100). The NO_2_^−^ concentration was determined using the Griess technique, which consists of detecting a color change of the solution to pink when NO_2_^−^ reacts sequentially with Griess reagents I and II (sulfanilic acid and α-naphthylamine). After adding the reagents, the sample was allowed to react for 20 min and the absorbance was measured at 520 nm. NO_3_^−^ measurements were performed by a reduction to nitrite with hydrazine. The NO_3_^−^ concentration was calculated as the difference between the nitrite concentration after and before reduction with hydrazine. H_2_O_2_ was measured using the peroxidase method, which catalyzes the reaction of H_2_O_2_ with 4-aminophenazone and phenol to give a red product (measured at 505 nm). Simultaneously, the corresponding calibration curves and their respective quality controls were processed to determine the reactive species. Analytical grade reagents were used (Cicarelli, Argentina). To evaluate the stability of PAW, physicochemical properties were measured at days 1, 7, 10, and 14 after activation.

### 2.3. Plasma-Activated Water Treatments

The PAW treatments applied in the germination and plant growth tests were tap-water-activated at different plasma exposure times. Tap water was obtained from a water well located in the city of Venado Tuerto (province of Santa Fe, in the central region of Argentina). The tap water was activated for 5, 10, or 15 min, and the treatments were named PAW5, PAW10, and PAW15, respectively. The control treatment consisted of non-activated tap water (C).

### 2.4. Germination Test

Tomato (*Solanum lycopersicum*) and bell pepper (*Capsicum annuum*) seeds were used for germination tests. The treatments used were plasma-activated tap water (PAW5, PAW10, and PAW15). The control treatment consisted of tap water (C). Six replicates of twenty-five seeds for each treatment were tested using the between-paper (BP) germination test proposed in the ISTA rules [24]. The test was conducted in a germination chamber (temperature: 20 <=> 30 °C, photoperiod 16/8 h (light/dark)). The germination percentage (G%) was reported as the proportion of seeds that produced normal seedlings 14 days after the start of the test [24].

### 2.5. Seeds Water Absorption

Ten dry seeds were placed in a Petri dish, and each seed was weighed (*m*_0_). Each plate contained two filter papers soaked with 3 mL of the corresponding treatment (PAW5, PAW10, and PAW15), whereas the control treatment was soaked in tap water (C). Seeds were removed from the plates and dried with paper towels, and each seed was weighed (*m_t_*) 1, 2, 5, 8, 24, and 30 h after the start of the test. The water absorption ratio at each time point was calculated as *m_t_*/*m*_0_. This coefficient was used to plot a curve showing the average time dependence of water absorption by the seeds. The test was performed in duplicates.

### 2.6. Plant Growth

Tomato and bell pepper seeds were sown individually in 1 L plastic pots filled with 120 g of GROWMIX^®^ MULTIPRO substrate (fine fiber Sphagnum moss peat, fine bark compost, perlite, and wetting agents, pH: 5–5.8, 250–450 ppm nitrate, 30–100 ppm phosphate, 200–300 ppm potassium). Five pots were used per treatment. The treatments included plasma-activated tap water (PAW5, PAW10, and PAW15), with tap water as the control (C). Pots were maintained in a growth chamber under controlled conditions: a 16/8 h light/dark photoperiod, day/night temperature of 25 ± 2 °C, and a photosynthetic photon flux density of 350 μE m^−2^s^−1^. Plants were irrigated according to crop demand, receiving approximately 100 mL of the corresponding treatment (or control) per irrigation event over 40 days. Irrigation solutions were poured directly onto the substrate. The water used for irrigation, both PAW and control, was renewed every 1–2 weeks, depending on plant demand and the growth stage. Importantly, PAWs and control water were not used beyond 14 days after preparation to ensure consistent treatment quality. The experiment was performed twice.

### 2.7. Biometric Parameters of the Plants

The fresh weight of the plants was measured using a balance with an appreciation error of ±1 g. The plants were then dried at 80 °C for 120 h, and their dry weight was recorded using an analytical balance with a precision of 0.001 g. The length of the aerial and root parts was determined by gently stretching each part to its maximum elongation and measuring it with a graduated ruler with 1 mm precision. Leaf chlorophyll content was assessed using an atLEAF CHL BLUE chlorophyll meter (FT Green LLC, Wilmington, DE, USA). Measurements were taken on the fourth true leaf of each plant, and chlorophyll content was expressed in arbitrary units (a.u.). Additionally, the number of leaves was recorded in bell pepper plants to provide further growth metrics.

### 2.8. Oxidative Stress of the Plants

#### 2.8.1. Lipid Peroxidation

Oxidative stress was assessed by measuring lipid peroxidation as malondialdehyde (MDA) content [25]. Fresh samples of the third true leaf were homogenized in a 20% (*w*/*v*) trichloroacetic acid (TCA) solution and centrifuged at 3500 g for 20 min. An aliquot of the supernatant (1 mL), 1 mL of 0.5% (*w*/*v*) thiobarbituric acid, and 100 µL of 4% butylated hydroxytoluene were added. The mixture was heated at 95 °C for 30 min and cooled on ice. It was centrifuged at 3000g for 15 min, and thiobarbituric acid-reactive substances (TBARS) were determined at 532 nm (A1), and unspecific absorbance was determined at 600 nm (A2). The TBARS concentrations were obtained by subtracting the absorbance (A1 − A2) and employing the MDA extinction coefficient (155 mM^−1^ cm^−1^). The results are expressed as nmol MDA (g tissue)^−1^. For each experimental repetition, five biological replicates for each treatment and two technical replicates for each sample were prepared.

#### 2.8.2. Antioxidant Enzymes

To prepare the extracts, 0.3 g of the third true leaf was taken and homogenized with 1 mg of polyvinylpyrrolidone (PVP) in 3 mL of 50 mM phosphate extraction buffer (pH 7.7), 0.5 mM EDTA, and 0.5% (*v*/*v*) Triton X-100. The homogenates were centrifuged for 30 min at 13,000× *g* and 4 °C. The supernatant fraction was used for all assays. The protein content of the extracts was determined according to the Bradford method of [26]. For each experimental repetition, five biological replicates for each treatment and two technical replicates for each sample were prepared.

For the determination of catalase (CAT), the reaction medium contained 100 μL of the homogenate, 50 mM potassium phosphate buffer (pH 7.2), and 2 mM H_2_O_2_. The activity was determined at 30 °C by measuring the decrease in absorbance at 240 nm due to the consumption of hydrogen peroxide. The pseudo-first-order reaction constant (*k*′ = *k* [CAT]) was determined from the decrease in H_2_O_2_ absorbance and the CAT content was calculated in pmol (mg of protein)^−1^ using a *k* = 4.7 × 10^7^ M^−1^ s^−1^ [27]. Superoxide dismutase (SOD) activity was measured by the inhibition of photochemical reduction by nitroblue tetrazolium (NBT), as described by [28]. The reaction mixture consisted of 200 μL of the supernatant and 3.5 mL of O_2_^−^ generating solution, containing 14.3 mM methionine, 82.5 μM nitro blue tetrazolium (NBT), and 2.2 μM riboflavine. The extracts were brought to a final volume of 0.3 mL with 50 mM potassium phosphate and EDTA (0.1 mM). The absorbance at initial time was determined at 560 nm, the test tubes were placed at 25 °C and illuminated with fluorescent tubes. The reaction was started and stopped by turning the light on and off (there was no detectable reaction variation under ambient light during reagent preparation and spectrophotometric measurements). The reduction in NBT was followed by absorbance reading at 560 nm every 2 min for 6 min. Identical tubes without enzyme extracts were used as blanks. One unit of SOD is defined as the amount of enzyme required to produce 50% inhibition of NBT reduction under assay conditions [29]. The SOD activity of the extracts was expressed as U (mg protein)^−1^. Guaiacol peroxidase (GPOX) enzyme activity was determined according to [30] by guaiacol oxidation measured at 470 nm (ε = 26.6 mM^−1^ cm^−1^) and expressed in μmol min^−1^ (mg protein)^−1^. The reaction mixture contained 50 mM potassium phosphate buffer (pH 7.2), 2 mM H_2_O_2_, 10 mM guaiacol, and 150 μL of plant extract. Two technical replicates were performed for each treatment.

### 2.9. Statistical Analysis

Statistical analyses were performed using the R software version 4.3.1 [31]. The variance of the data was analyzed using one-way analysis of variance (ANOVA). After checking the assumptions of normality of the residuals, homogeneity of variances, and independence of observations, the Fisher test (LSD test) was performed. Differences were considered statistically significant at *p* < 0.05. All data are shown as the mean ± standard error of the mean.

## 3. Results

### 3.1. Physicochemical Properties of Plasma-Activated Water

**Figure 3:**
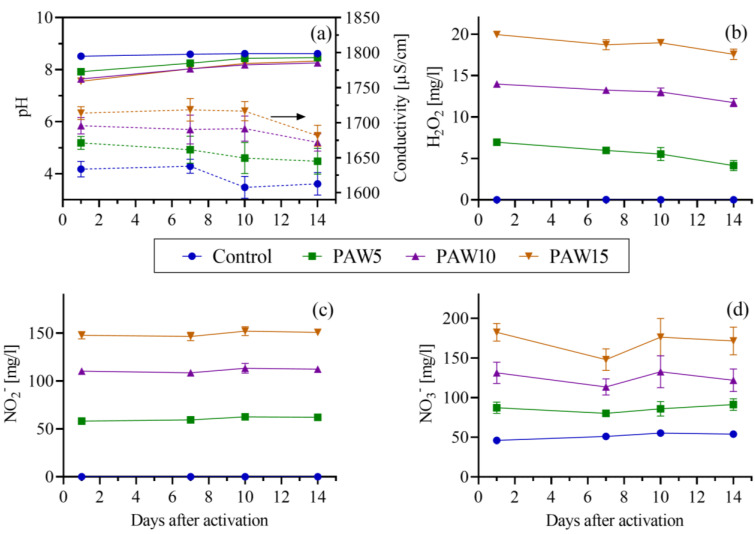
Physicochemical properties of PAW up to 14 days after activation: pH (solid lines, left axis) and electrical conductivity (dashed lines, right axis) (**a**); and aqueous phase concentrations of hydrogen peroxide (**b**), nitrite (**c**), and nitrate (**d**). The arrow indicates that the axis corresponding to electrical conductivity is the one on the right. Therefore, the axis on the left corresponds to pH. The dashed lines correspond to electrical conductivity.

In Figure 3, the physicochemical properties of the PAW are shown. It can be observed that, as the water exposure time to plasma increased, the electrical conductivity increased and the pH decreased (Figure 3a). Increased electrical conductivity and decreased pH are characteristic of water in contact with non-thermal discharges in air or similar mixtures. This is related to the formation of acids (HNO_3_ and HNO_2_) and ions (mainly H^+^, NO_2_^−^, and NO_3_^−^) in the solution [32]. However, the decrease in pH was slight and the liquid remained around a neutral pH. This slight decrease in pH is due to the buffer system of tap water given by the hydrocarbon of the water [33]. Regarding the concentration of RONS, it was observed that tap water (C) contained only NO_3_^−^ (the concentrations of H_2_O_2_ and NO_2_^−^ were below the detection limit of the techniques), while the PAW contained H_2_O_2_, NO_2_^−^ and NO_3_^−^ (Figure 3b–d). The concentrations of the three reactive species increased with activation time, reaching their maximum values after 15 min of treatment: 20 mg/L of H_2_O_2_, 147.6 mg/L of NO_2_^−^, and 182.3 mg/L of NO_3_^−^. These RONS concentrations are significantly higher than those reported in previous studies on the effects of PAW on tomato and pepper [14,15,16,17,18,19,20]. Specifically, the H_2_O_2_ concentration was between 4 and 200 times greater [14,15,16,17,20], while the RNS (NO_X_ = NO_2_^−^ + NO_3_^−^) concentration (=5.4 mM) was 3–22 times higher. Figure 3 further illustrates that the physicochemical properties of the PAW remained relatively stable for up to 14 days. This stability is consistent with the behavior of PAW at a neutral pH, as aqueous kinetics are more reactive at lower pH levels [34].

Considering that non-thermal plasmas are a method of RONS synthesis in liquids [7], an important parameter to evaluate is the energy efficiency of the RONS synthesis process. The average energy efficiency can be estimated as *η* = *C_i_*/*ε*, where *C_i_* is the aqueous phase concentration of RONS of species *i* (in [mol/L]), and *ε* is the mean energy delivered to the discharge per liter of water (in [J/L]) [21]. This coefficient allows a comparison of the efficiency of the RONS synthesis process regardless of the type of discharge used, discharge power, volume of water activated, and activation time. Table 1 shows the average energy efficiency of ROS (H_2_O_2_) in water in contact with the non-thermal discharges applied to tomato and pepper irrigation [14,15,17,18,19,20], as well as for discharges such as the one used in this work [21,35]. The average energy efficiency in H_2_O_2_ synthesis in the liquid volume obtained in this work is fairly high, between 3 and 60 times that reported in the rest of the papers.

**Figure 4:**
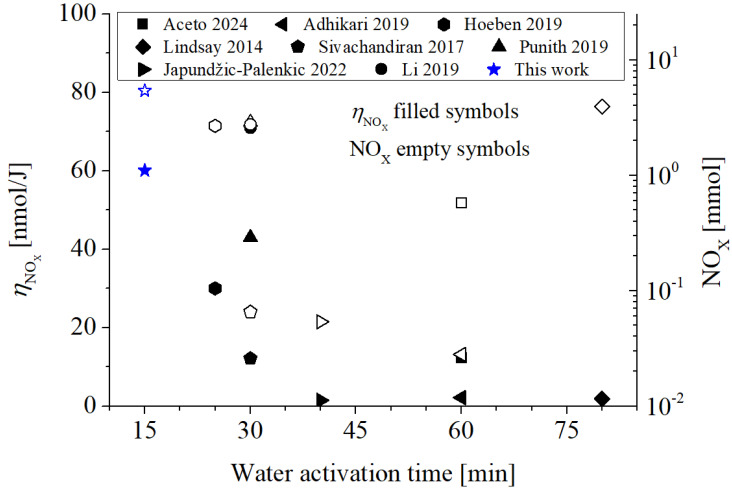
Average energy efficiency for RNS (NO_X_) synthesis, *η*_NOx_ (left axis), and mass of NO_X_ generation in the liquid (right axis). The corresponding experimental data are: Aceto 2024 [14], Adhikari 2019 [15], Japundžić-Palenkić 2022 [17], Lindsay 2014 [18], Punith 2019 [19], Sivachandiran 2017 [20], Hoeben 2019 [21], Li 2019 [35]. The hollow symbols (in this case the hollow star) correspond to the mass of RNS generated in the liquid.

Figure 4 shows the average energy efficiency for the synthesis of RNS (NO_X_ = NO_2_^−^ + NO_3_^−^) corresponding to the data given in Figure 3c,d for PAW15 at one day after activation (147.6 mg/L for NO_2_^−^, and 136.8 for NO_3_^−^). In calculating the average energy efficiency for NO_3_^−^, the initial nitrate concentration (45.9 mg/L) in the control was subtracted from the corresponding value after activation (182.3 mg/L). It can be observed that the average energy efficiency of RNS synthesis of this work is among the highest (60 nmol/J), only below the efficiency obtained in [35]. The highest average energy efficiencies are those corresponding to glow-type (or arc) discharges with low power (≲100 W; e.g., [19,35] and this study) rather than DBD or jet-type discharges.

Figure 4 also shows the mass of NO_X_ (=NO_2_^−^ + NO_3_^−^) (calculated as *C*_NOx_ × *volume*) produced in water (right axis, empty symbols). The highest amount of NO_X_ produced in water is reported in this work, with a value of ≈5.4 mmol. The studies that follow are those reported by [18,19,21,35], with values between 25 and 45% lower (3–4 mmol of NO_X_). It is important to highlight that the amount of NO_X_ in water achieved in this work was obtained for a relatively high volume and in a time of only 15 min: most of the studies (Table 1) activate volumes ≤ 0.5 L (with the exception of [18,19], with volumes of ≈2 L) for significantly longer times (from 25 to the 80 min) than required in our work.

### 3.2. Seeds Germination and Water Absorption

**Figure 5:**
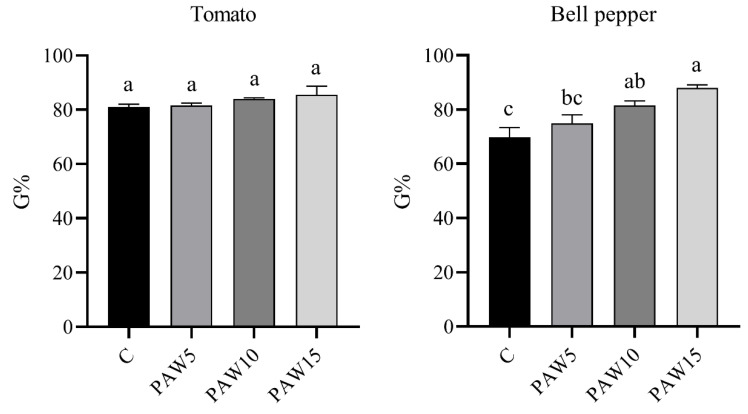
Germination percentage (G%) of tomato (**left**) and bell pepper (**right**) seeds 14 days after the start of the test. Different letters denote statistical differences (one-way ANOVA, LSD post-test, *p* < 0.05).

The germination percentage (G%) was measured 14 days after the start of the experiment for both tomato and bell pepper seeds irrigated with PAW and non-activated water (C), as shown in Figure 5. For the tomato seeds, the control had a G% of 80%, whereas a slight increase was observed in the seeds irrigated with PAW. However, no significant differences were found between the PAW-treated and control seeds. In contrast, for bell pepper seeds, a significant increase in G% was observed compared with the control. G% increased by 7%, 17%, and 26% for PAW5, PAW10, and PAW15, respectively, compared with the control, which had a G% of approximately 70%. Overall, no negative effects on germination were observed for either crop irrigated with PAW compared to the control.

**Figure 6:**
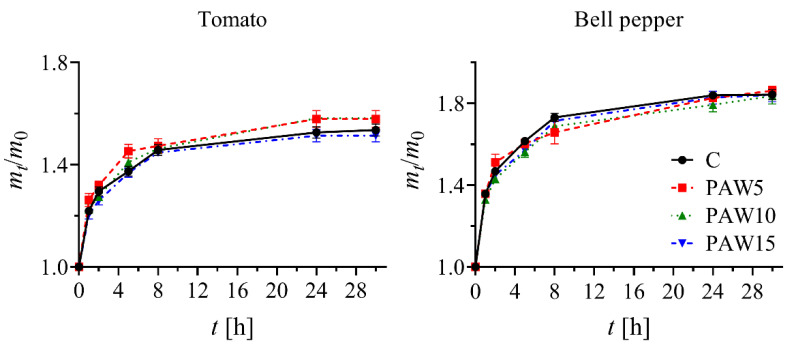
Water absorption of tomato (**left**) and bell pepper (**right**) seeds up to 30 h of imbibition.

Figure 6 illustrates the water absorption of tomato and bell pepper seeds exposed to PAW for 30 h. Although differences in water absorption can be observed (higher values with PAW5 and PAW10 than with C and PAW15 in tomato, and higher values with C than with PAW treatments in bell pepper), these differences are not statistically significant. The results showed no significant effect of PAW on water absorption compared to the control, for any crop. However, the seeds exposed to PAW have been reported to undergo modifications in their surface structures [4,8,36,37], and under these conditions, it is possible that water absorption is different from that of seeds not treated with PAW. Additionally, scanning electron microscopy (SEM) images were taken to evaluate the potential changes in the surface structure of tomato and bell pepper seeds after 24 h of PAW treatment. In tomato seeds, glandular trichomes completely covered the surface, preventing its observation. In bell pepper seeds, no differences in the surface structure were observed. These findings support the observation that PAW treatment does not alter seed water absorption, at least not through changes in surface structure.

### 3.3. Plant Growth

**Figure 7:**
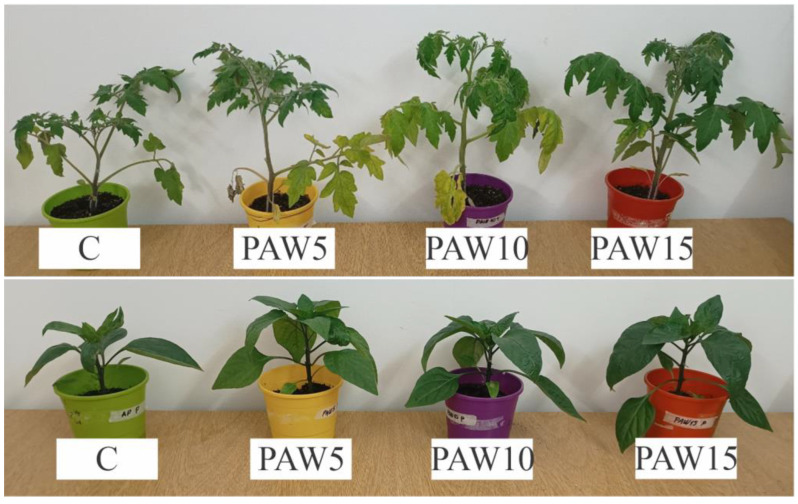
Tomato (**above**) and bell pepper (**bottom**) plants 40 days after sowing.

Figure 7 show tomato and bell pepper plants at harvest (40 days after sowing). At first glance, PAW-irrigated plants did not show any damage compared to the control plants. It is worth noting that the plants, even those irrigated with the control, were not subjected to nutrient deficiency because they grew in a nutrient-rich substrate (see Section 2.6. in Materials and Methods), which is typical for this type of crop that grows in controlled greenhouse environments [14]. Some differences in plant size and leaf coverage were observed between plants treated with PAW and the control plants. A significant increase in the number of leaves was observed in bell pepper plants (one-way ANOVA, *p* < 0.05): control 15 ± 1, PAW5 17 ± 1, PAW10 19 ± 1, and PAW15 24 ± 1. This represents an increase in the number of leaves between 13 and 60% in bell pepper plants. This result agrees with those reported by other authors, who evaluated the effect of PAW on the leaf cover of tomato and pepper plants [14,16,18,19] as well as on other types of plants [38,39]. For both the quantitative and qualitative results, the effect of PAW on plant leaf cover was significant.

**Figure 8:**
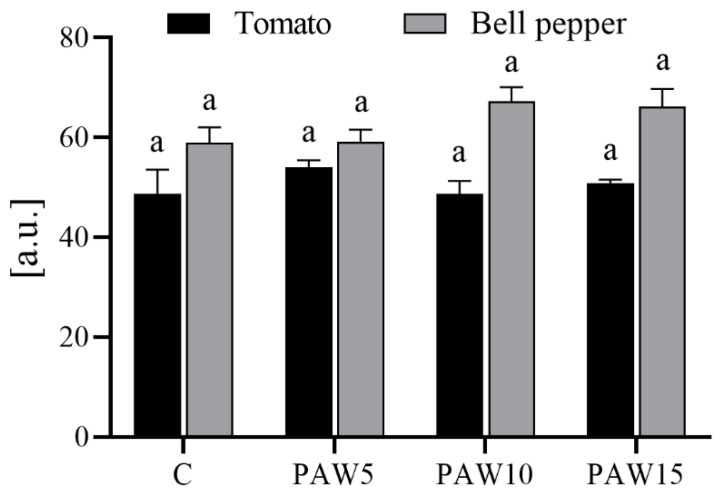
The total chlorophyll content of the leaves in tomato and bell pepper plants at 40 days after sowing as a function of the treatments. The determination was made on the fourth true leaf. Different letters denote statistical differences (one-way ANOVA, LSD post-test, *p* < 0.05).

No differences were observed in the chlorophyll contents of tomato and bell pepper plants irrigated with PAW compared to those irrigated with the control treatment (Figure 8). In the case of tomato, a slight increase in chlorophyll content was observed in plants irrigated with PAW5 compared to the other treatments and the control, although there were no significant differences between them. For bell pepper, plants irrigated with PAW10 and PAW15 showed an increase in chlorophyll content compared to plants treated with PAW5 or the control, although the differences were not significant.

**Figure 9:**
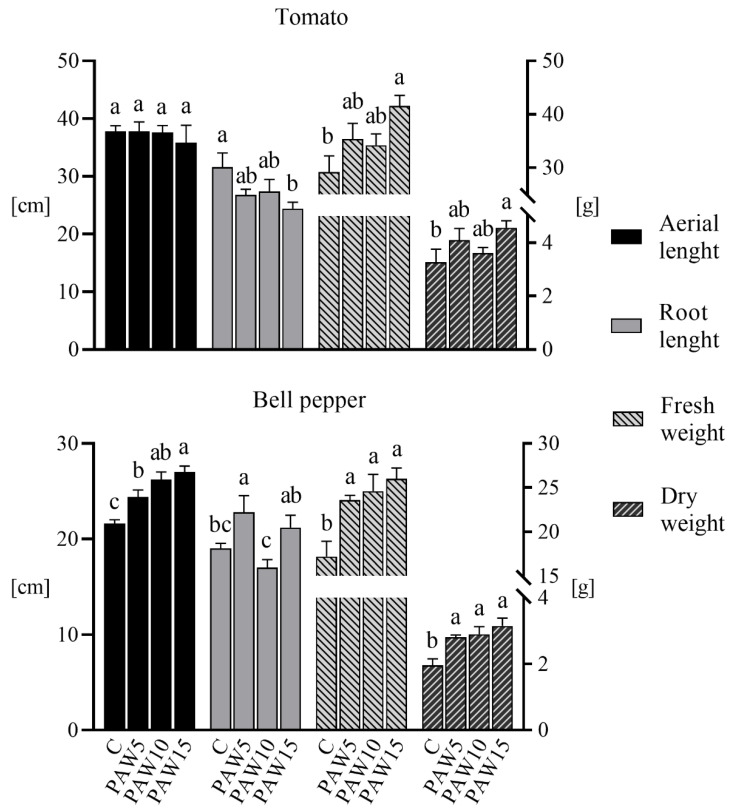
Biometric parameters (aerial and root lengths and fresh and dry weights) of the tomato (**above**) and bell pepper (**bottom**) plants as a function of the treatments. Different letters denote statistical differences (one-way ANOVA, LSD post-test, *p* < 0.05).

Figure 9 shows the effect of PAW on the biometric parameters evaluated (aerial and root lengths, and fresh and dry weights) in tomato and bell pepper plants 40 days after sowing. For tomato, no difference in the aerial length of plants irrigated with PAW compared to the control was observed, whereas the root length decreased as the PAW activation time increased (15% for PAW5 and PAW10, and 23% for PAW15 with respect to the control). Regarding the roots of the tomato plants, it is noteworthy that, while the root length decreased with increasing PAW activation time, a significant increase in the root dry weight was observed (one-way ANOVA, *p* < 0.05). Dry weight values (in grams) were as follows: control 0.97 ± 0.14, PAW5 1.27 ± 0.06, PAW10 1.22 ± 0.09, and PAW15 1.57 ± 0.09. This represents an increase in root dry weight of 25% to 62%. The fresh weight of tomato plants irrigated with PAW increased significantly with respect to the control (approximately 20% for PAW5 and PAW10, and 42% for PAW15), whereas in PAW-irrigated plants, dry weight increased by 11–39% compared to control plants.

Figure 9 also shows that, for bell pepper plants, the aerial length significantly increased with PAW irrigation compared to that of the control group (13% increase for PAW5, 21% for PAW10, and 25% for PAW15). However, no clear effect or trend was observed in the root length of PAW-irrigated plants relative to that of the control. In terms of fresh weight, bell pepper plants irrigated with PAW had significantly higher weights than plants irrigated with the control (increases of 37, 43, and 51% for PAW5, PAW10, and PAW15, respectively). The dry weight of plants irrigated with PAW also showed a significant increase compared to control plants: 44, 48, and 61% for PAW5, PAW10, and PAW15, respectively.

### 3.4. Oxidative Stress of the Plants

**Figure 10:**
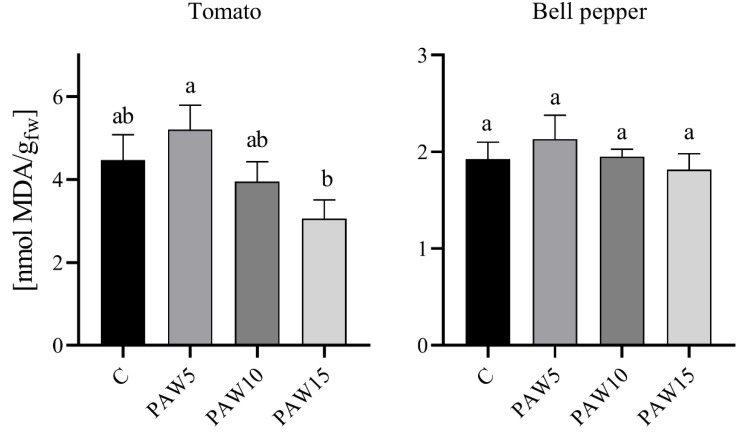
Effect of plasma-activated water on lipid peroxidation as the malondialdehyde (MDA) content of plants of tomato and bell pepper 40 days after sowing. Different letters denote statistical differences (one-way ANOVA, LSD post-test, *p* < 0.05).

Figure 10 shows that plants irrigated with PAW had a similar MDA content in leaves as the control plants. MDA is a by-product of lipid oxidation in plants and is therefore often used as an indicator of cell damage caused by RONS. The results showed no significant differences between the treatments and the control for both crops. A small increase was observed in plants irrigated with PAW5 for both tomato and bell pepper, although this trend changed for plants irrigated with PAW10 and PAW15, where the MDA content was similar to or lower than that of the control plants. Based on these results, it can be said that the PAWs evaluated in our study do not present an oxidative toxicity effect on plants. In [15], the MDA content in tomato plants irrigated for 35 days with activated water for 15, 30, or 60 min was quantified. Their results showed that, in the aerial parts, plants irrigated with PAW15 and PAW30 presented less damage than the control plants; however, plants irrigated with PAW60 had a higher MDA content. The authors postulated that this increase in MDA content is due to the toxic effect of PAW due to its high concentration of RONS.

Table 2 shows the activities of the antioxidant enzymes CAT, SOD, and GPOX in the aerial part of the plants. For tomato, the CAT activity increased significantly with increasing PAW activation time, while SOD activity showed a tendency to decrease with the water activation time, without a significant differences compared to the control plants. The highest GPOX activity was observed in plants irrigated with PAW5, whereas those irrigated with PAW10 and PAW15 showed activity levels that were not different from those of the control. The enzyme activities of the bell pepper plants irrigated with PAW showed no statistical differences from those of the control plants. However, it is interesting to note that the CAT activity increased as a function of the PAW activation time; SOD and GPOX showed their highest activities in plants irrigated with PAW5, while those irrigated with PAW10 and PAW15 showed activity levels like those of control plants.

## 4. Discussion

### 4.1. Physicochemical Properties of Plasma-Activated Water

The high values of H_2_O_2_ concentration and average energy efficiency for the H_2_O_2_ synthesis (6.5 nmol/J, Table 1) achieved in this work is probably related to the cooling through refrigerated water bath system and mixing through the vortex (see Figure 1) during the activation process, which avoids the excessive heating of the PAW and the consequent thermal destruction of H_2_O_2_, as postulated by the work of [21]. The highest levels of the other studies (≈2 nmol/J, Table 1) were achieved by [14,21]. Although in [14] the PAW was not cooled during the activation process, given the low discharge power and the relatively high-activated volume (0.5 L), it is likely that the solution temperature was not raised high enough to thermally dissociate the H_2_O_2_ molecule.

As expected, the highest values of NO_X_ concentration and energy efficiencies of NO_X_ (RNS) synthesis (Figure 4) correspond to discharges with a high gas temperature (i.e., glow-type discharges) rather than DBD or jet-type discharges. In glow-type discharges burning in air (or similar mixtures) where the discharge gas temperature is high (of the order of 1000 K), the endothermic oxidation of the air takes place in the recombination zone [21].

NO molecules are the primary precursors for NO_X_ formation, primarily produced via Zeldovich reactions. The rate of NO formation increases significantly with higher discharge gas temperatures, with a difference of several orders of magnitude observed for a ten-fold increase in temperature [34]. In this study, the discharge temperature (~3500 K) was sufficiently high to generate appreciable NO concentrations (in the order of 10^22^–10^23^ m^−3^) [40], leading to elevated NO levels in the gaseous recombination zone.

In a humid air atmosphere, NO undergoes oxidation to form NO_X_ (mainly NO_2_ and NO_3_) and acids (HNO_2_ and HNO_3_). Despite the high NO concentration in the recombination zone, its solubility in water is relatively low compared to species like NO_2_, HNO_2_, and HNO_3_. Consequently, the formation of NO_3_^−^ and NO_2_^−^ in the aqueous phase is primarily attributed to the transfer of NO_2_ and acidic species from the gas phase into the liquid [34].

Regarding the gas-to-liquid transfer, some authors suggest that this occurs primarily through the discharge, specifically via the cathodic root [41]. However, diffusion through the gas–liquid interface in the recombination zone is also expected, particularly in this study, where the confined gas chamber prevents RONS from escaping into the environment [42]. Additionally, the gas–liquid interface in the recombination zone is substantially larger than the plasma–liquid interface.

The vortex effect further enhances this transfer by disrupting the boundary layer at the liquid interface, which would otherwise form under static conditions. This combination of a confined gas chamber and vortex-induced mixing likely promotes greater RONS diffusion from the gas phase to the liquid phase, enhancing the overall efficiency of the process.

The average energy efficiency of the NO_X_ synthesis of the present our work is only below the efficiency obtained in [35]. This can be attributed to the fact that the power consumed in that study is lower than ours, and therefore a larger part of the energy delivered to the discharge is directed to the generation of RNS in the recombination zone instead of being transferred to gas heating. This would explain the results of [18], where the power is 420 W and the energy efficiency of RNS synthesis is low (~1 nmol/J).

### 4.2. Seed Germination, Plant Growth, and the Oxidative Stress of the Plants

When seeds are exposed to the PAW obtained through discharges in air or similar mixtures, they are subjected to an aqueous solution containing significant amounts of RONS, particularly NO_3_^−^, NO_2_^−^, and H_2_O_2_. Changes in the composition of RONS across the different PAW treatments are shown in Figure 3. RONS can induce both physical and physiological changes in the seeds [4,6]. In recent years, the effects of PAW on germination have been studied in various seed types [10,16,20,36,37,43,44]. For example, in [16], aged pepper seeds irrigated with PAW showed a 28.3% increase in germination after eight days compared to the control. These results are consistent with our findings for bell peppers (Figure 5), in which we observed a direct correlation between the increase in G% and the proportional increase in NO_3_^−^, NO_2_^−^, and H_2_O_2_ concentrations as a function of plasma exposure time (Figure 3). This is in line with [10], who postulated that reactive species, such as H_2_O_2_ and NO_3_^−^, are responsible for the increase in germination, particularly through their synergistic effect in breaking seed dormancy by promoting the endogenous production of NO radicals.

The results showed that PAW significantly stimulated the growth of tomato and bell pepper plants (Figure 9), with growth increasing as the water activation time (and RONS content) increased. PAW contains mainly NO_3_^−^ and NO_2_^−^ (Figure 3), which are key species for plant nutrition. However, there were differences in the response of each crop to the effect of PAW: the most marked effects on growth parameters were obtained for bell pepper plants, with the major increases in aerial length and fresh and dry weights, and corresponding with the germination results, where the most significant improvements were also obtained for bell pepper seeds (Figure 5). The results of the biometric parameters of the plants are in line with those reported in another study [20], where the effect of PAW on the growth of tomato and pepper plants was also evaluated. Their results also showed that the most pronounced effects on plant growth were obtained for pepper, indicating that the effect of PAW depends on the type of crop.

In tomato plants, the observation that the roots of PAW-irrigated plants are shorter but heavier than those of control plants might seem contradictory at first. This discrepancy arises from the methodology used for root length measurement (Section 2.7), which involved softly stretching the roots to their maximum extension. It is important to note that, while root length is often reported as an indicator of plant development, some researchers caution against its use due to its sensitivity to external factors such as light quality and temperature differentials. Instead, they recommend assessing plant development through biomass measurements, particularly dry weight, as it is a more reliable indicator of growth, reflecting carbon fixation [4,33]. In this study, although tomato PAW-irrigated plants had shorter roots, their increased biomass—evidenced by a higher dry weight—provides a more accurate representation of enhanced root development.

The remaining works that evaluated the influence of PAW on tomato and pepper also found a stimulating effect on plant growth. Regarding tomato plants, an increase of ≈30% in stem length after 21 days was observed in [14] for plants irrigated with PAW, relative to the positive (nutrient solutions) and negative (distilled water) controls evaluated. The work of [18] obtained significant increases (between 1.7 and 2 times) in the dry weight of the aerial parts of plants irrigated with PAW with respect to the control. In [19], significant increases were found in the stem length (≈1.5-fold) and wet weight (up to 61%) of 46 days-old plants irrigated with activated tap water for 30 min, compared to those irrigated with non-activated water. For pepper plants, [16] reported increases of up to 138.6% in root length, up to 67.6% in stem length, and up to 194.4% in the fresh weight of pepper plants irrigated with PAW relative to the control. As can be seen, the results of our work and other authors show the stimulatory effect of PAW on plant growth. However, the influence of PAW also depends on many factors (type of discharge, type of activated solution—distilled water, tap water, or a nutrient solution—type of soil or substrate, the experimental conditions); therefore, it is difficult to predict the effect of PAW on plant growth [20,33].

As mentioned earlier, PAW is a solution with a high content of RONS, which serve as key components for plant nutrition and act as signaling molecules that regulate plant growth. However, some authors have been unable to evaluate the effect of PAW on the development of plants grown in nutrient-rich soils because the PAW effect was negligible [45]. This is probably due to the low RONS content of the solution. In this regard, in our experiment, the plants were grown on a nutrient-rich substrate, which is typical for this type of crop [14], and despite this, the effect of PAW on plant development was significant.

The ability of plants to withstand oxidative stress largely depends on their capacity to upregulate antioxidant enzyme activity [45]. The presence of RONS in PAW is expected to influence this enzymatic response. SOD converts superoxide radicals into H_2_O_2_, while CAT transforms H_2_O_2_ into water and oxygen and GPOX uses H_2_O_2_ to oxidize substrates. Previous studies have shown that PAW irrigation increases endogenous H_2_O_2_ and NO_X_ levels in tomato seedlings as the water activation time increases [15]. Consistent with these findings, the endogenous RONS content in PAW-irrigated plants was probably also increased in our study. In barley seedlings, for instance, elevated RONS levels were linked to increased SOD and CAT activity, a mechanism attributed to the induction of antioxidant enzyme expression to detoxify ROS and maintain redox balance [46]. This mechanism aligns with our findings, where CAT activity increased in both crops with increasing PAW activation time, as well as the heightened GPOX activity in tomatoes and SOD and GPOX activities in bell pepper plants irrigated with PAW5. Notably, lipid peroxidation (MDA content, Figure 10) showed no significant differences between PAW-treated and control plants, reinforcing that the antioxidant defense system effectively mitigated oxidative damage. Interestingly, the observed decrease in SOD activity with an increasing PAW activation time aligns with findings in lettuce plants, where the presence of NO_3_^−^ in PAW was postulated to influence this response [33]. Overall, the results indicate that the antioxidant system (CAT, SOD, and GPOX) in PAW-irrigated plants successfully metabolized RONS, preventing cellular damage and demonstrating the potential of PAW as a sustainable irrigation strategy.

Other authors have also evaluated the effects of enzymes or antioxidant molecules in plants irrigated with PAW. The paper of [14] evaluated the effect of PAW on antioxidant molecules in tomato plants and their results showed that PAW increased the ascorbate (vitamin C) content, suggesting that PAW enhances the plant defense system. In [15], the expressions of *sod* and *cat* in the aerial parts of tomato plants were determined. The authors found that the overexpression of the *sod* in plants irrigated with PAW were activated for 30 min, and underexpression or similar levels were observed for control plants and for those irrigated with PAW activated for 15 or 60 min, respectively. For the *cat*, plants irrigated with 15 min PAW showed no differences compared to the control, whereas those irrigated with 30 and 60 min PAW showed overexpression. It is worth noting that, despite the overexpression of the *cat* in plants irrigated with 60 min PAW, oxidative damage (MDA) was not compensated, unlike in plants irrigated with 30 min PAW, where the plant defense system was able to avoid oxidative damage. In the work of [16], the CAT and SOD activities in the roots of pepper plants irrigated with PAW were determined. Their results showed an increase in CAT activity in plants irrigated with PAW relative to the control, and similar levels of SOD activity between the treatments and the control. In [45], the activities of CAT, SOD, and GPOX in the aerial parts of wheat plants grown on perlite (nutrient-free substrate) were evaluated for four weeks. Their results showed that the activities of enzymes irrigated with PAW decreased compared to the control (tap water), although they were higher than those of plants irrigated with the nutrient solution. It is expected that enzyme activities in control plants will be higher than those in the treatments, since these plants are facing higher stress state due to the lack of available nutrients.

## 5. Conclusions

In the present study, the effect of PAW on the germination and growth of tomato and bell pepper plants was evaluated, in addition to the oxidative stress and antioxidant enzyme activity of the plants. PAW was obtained from tap water using a 1 L capacity plasma reactor based on a quasi-stationary (50 Hz) glow-type discharge in atmospheric pressure air with a water-cathode. The results showed the following:

1. The long-lived RONS concentrations obtained in PAW after only 15 min of activation were significantly high: 20 mg/L of H_2_O_2_, 147.6 mg/L of NO_2_^−^, and 182.3 mg/L of NO_3_^−^. These RONS concentrations are significantly higher than those reported in previous studies on the effects of PAW on tomato and pepper. Specifically, the H_2_O_2_ concentration was between 4 and 200 times greater, while the RNS (NO_X_ = NO_2_^−^ + NO_3_^−^) concentration (=5.4 mM) was 3 to 22 times higher.
2. The average energy efficiency of RONS synthesis in the water of the plasma reactor was among the highest compared to those reported in similar studies: 60 nmol/J for NO_X_ synthesis and 6.5 nmol/J for H_2_O_2_ synthesis.
3. The effect of PAW was more significant in bell pepper than in tomato: the greatest increases in both germination and plant biometric parameters were obtained for bell pepper.
4. PAW promoted the germination of bell pepper seeds, with an increase of up to 26% in the germination percentage at 14 days. No effect of PAW on tomato seed germination was observed.
5. PAW significantly increased the plant growth, even in a nutrient-rich substrate: the bell pepper fresh weight increased between 37 and 51% and the dry weight between 44 and 61%—while the corresponding increases for tomato were between 20 and 42% in fresh weight and between 11 and 39% in dry weight.
6. The PAWs evaluated did not induce oxidative stress in the plants, as evidenced by the similar levels of MDA content observed in PAW-irrigated and control plants. Additionally, tomato and bell pepper plants irrigated with PAW exhibited SOD and GPOX activities comparable to those of the control plants. Interestingly, CAT activity increased with PAW activation time.

PAW is presented as an innovative alternative for crop nutrition through irrigation, one that it is eco-friendly because it does not require chemicals compounds, is capable of promoting germination, and stimulates plant growth and defense system.

## Figures and Tables

**Table 1:** Average energy efficiency of H_2_O_2_ synthesis in water for different water contact discharges.

Reference	Discharge Type	Power [W]	Activation Time [min]	Volume [L]	*η* H_2_O_2_ [nmol/J]
[14]	DBD	13	60	0.5	2
[15]	Jet	3.6	60	0.05	0.1
[17]	Jet	15	40	0.215	0.7
[18]	Glow	420	80	1.9	-
[19]	Arc	37	30	2	-
[20]	DBD	3	30	0.25	0.1
[21]	Arc	150	25	0.4	2.1
[35]	Glow	22	30	0.4	0.8
This work	Glow	100	15	1	6.5

For the average energy efficiency *η* H_2_O_2_ of this work, the H_2_O_2_ concentration of PAW15 corresponds to that measured one day after activation (=20 mg/L = 0.588 mM, Figure 3b).

**Table 2:** Effect of plasma-activated water on CAT, SOD, and GPOX activities in the plants of tomato and bell pepper 40 days after sowing.

Crop	Treatment	CAT	SOD	GPOX
		[pmol/mg_prot_]	[U/mg_prot_]	[µmol/min/mg_prot_]
Tomato	C	2.2 ± 0.4 c	167.2 ± 52.0 a	123.1 ± 30.0 b
	PAW5	5.3 ± 1.2 c	142.9 ± 34.5 a	226.8 ± 39.3 a
	PAW10	9.5 ± 1.8 b	132.5 ± 34.3 a	94.6 ± 13.6 b
	PAW15	13.9 ± 1.0 a	86.7 ± 14.5 a	98.8 ± 17.0 b
Bell pepper	C	51.8 ± 1.3 a	68.5 ± 10.5 a	81.3 ± 18.6 a
	PAW5	55.7 ± 7.5 a	78.5 ± 11.7 a	133.2 ± 35.6 a
	PAW10	61.9 ± 6.5 a	60.4 ± 7.1 a	64.5 ± 16.9 a
	PAW15	66.0 ± 6.5 a	49.1 ± 2.9 a	72.1 ± 13.9 a

Different letters denote statistical differences (one-way ANOVA, LSD test, *p* < 0.05).

## Data Availability

The data supporting the findings of this study are available upon reasonable request from the authors.
